# Efficacy of gemcitabine and cetuximab combination treatment in head and neck squamous cell carcinoma

**DOI:** 10.3892/mco.2013.159

**Published:** 2013-07-24

**Authors:** SHINICHIRO MASEKI, KEI IJICHI, HAYAO NAKANISHI, YASUHISA HASEGAWA, TETSUYA OGAWA, SHINGO MURAKAMI

**Affiliations:** 1 Department of Otolaryngology-Head and Neck Surgery, Nagoya City University Graduate School of Medical Sciences, Nagoya, Aichi 467-8601, Japan; 2 Division of Oncological Pathology, Aichi Cancer Center Research Institute, Aichi 464-8681, Japan; 3 Department of Head and Neck Surgery, Aichi Cancer Center Hospital, Nagoya, Aichi 464-8681, Japan; 4 Department of Otolaryngology, Aichi Medical University School of Medicine, Nagakute, Aichi 480-1195, Japan

**Keywords:** head and neck squamous cell carcinoma, chemotherapy, cetuximab, gemcitabine, cell cycle

## Abstract

Head and neck squamous cell carcinoma (HNSCC) may be curable with surgery, radiation and chemotherapy in its early stages. However, recurrence and metastasis often prevail following primary treatment in advanced stage cases and are associated with significant morbidity and mortality. In this study we investigated the combination therapy of gemcitabine and cetuximab for HNSCC. The UM-SCC-6 and UM-SCC-23 HNSCC cell lines were analyzed following treatment with gemcitabine and cetuximab. To determine the mechanism of action of this combination treatment, the cell cycle distributions following gemcitabine and/or cetuximab treatment were analyzed by flow cytometry and apoptosis assay. Gemcitabine and cetuximab combination treatment exerted an enhanced cytotoxic effect. The cell cycle analysis demonstrated that cells accumulated in the S phase following gemcitabine treatment and G1 arrest occurred following cetuximab treatment. An increase in sub-G1 phase cells was also observed following treatment with the two drugs. In an apoptosis assay, caspase 3/7 activity was found to be higher when administering a combination of gemcitabine and cetuximab compared to each agent administered alone. Gemcitabine and cetuximab are individually effective against HNSCC and an enhanced growth inhibitory effect may be expected when these agents are used in combination.

## Introduction

Head and neck squamous cell carcinoma (HNSCC) may be curable with surgery, radiation and chemotherapy in its early stages. However, recurrence and metastasis often prevail following primary treatment in advanced stage cases and are associated with significant morbidity and mortality (1,2).

Chemotherapy with cisplatin and fluorouracil (5-FU) (PF regimen) has been widely used for HNSCC. Previous studies reported that the combination of 5-FU, cisplatin and docetaxel (TPF regimen) is more effective and more widely used compared to the PF regimen (3–5). However, the TPF regimen did not achieve notable decreases in recurrence and metastasis in advanced-stage cases. In addition, in cases of residual carcinoma and recurrence, the options of chemotherapeutic drugs are limited.

Epidermal growth factor receptor (EGFR) expression, as detected by immunohistochemistry, is present in >90% of HNSCC specimens and is associated with worse survival and locoregional failure (6). Agents that target EGFR have demonstrated its involvement in HNSCC. Cetuximab, a monoclonal antibody, was approved by the Food and Drug Administration for use in conjunction with radiation therapy for locally advanced HNSCC and as a single agent for incurable recurrent and metastatic disease (7).

Several mechanisms that contribute to the antitumor activity of cetuximab have been identified. A dominant mechanism is the interference of the antibody with the binding of natural ligands, such as epidermal growth factor and transforming growth factor to the EGFR receptor, thereby disrupting the EGFR signaling pathways (8). Another mechanism, the construction of cetuximab on an immunoglobulin G1 framework, may theoretically enable this agent to mediate antibody-dependent cellular cytotoxicity via natural killer cells and macrophages (9). Cetuximab also arrests the cell cycle in the G1 phase to induce apoptosis (10). Previous studies investigated the use of cetuximab in HNSCC in combination with cisplatin (11), radiation therapy (RT) (7), cisplatin + RT (12) and TPF (13). However, these drugs are the current option for primary treatment and potentially lead to acquired resistance to chemotherapy and recurrence or residual disease.

Gemcitabine (2′,2′-difluoro-2′-deoxycytidine) is a deoxycytidine analog with clinical activity in solid tumors, including pancreatic carcinoma, carcinoma of the biliary tract, non-small-cell lung carcinoma and head and neck cancer. Investigations of the antitumor activity of gemcitabine demonstrated that it is intracellularly activated by phosphorylation to gemcitabine triphosphate, which then interferes directly with DNA synthesis in tumor cells through the inhibition of DNA polymerization and incorporation of the fraudulent nucleotide into the growing DNA strand. Gemcitabine may also affect DNA synthesis by preventing the *de novo* biosynthesis of the deoxyribonucleoside triphosphate precursors through the inhibition of ribonucleotide reductase (14). In addition, gemcitabine causes S-phase accumulation. Therefore, gemcitabine is considered to be a radiosensitizer (15,16). There have been several phase II studies on gemcitabine + RT (17), gemcitabine + cisplatin (18) and gemcitabine + docetaxel (DTX) (19), which demonstrated that gemcitabine is effective against HNSCC.

Furthermore, there have been studies on the use of gemcitabine + cetuximab, such as a multicentre phase II study investigating the use of cetuximab + gemcitabine/oxaliplatin in metastatic pancreatic cancer (20). The synergistic effects of gemcitabine and gefitinib for the treatment of HNSCC have been demonstrated *in vitro*(21). However, it is hypothesized that cetuximab rather than gefitinib is to be clinically applied for the treatment of HNSCC in the future. Consequently, we investigated the combination therapy of gemcitabine and cetuximab for the treatment of HNSCC.

## Materials and methods

### Reagents

Gemcitabine was provided by Eli Lilly and Co. (Indianapolis, IN, USA). Cetuximab was provided by Merck Serono (Darmstadt, Germany).

### Cells and cell culture

The UM-SCC-6, UM-SCC-23 and UM-SCC-81B HNSCC cell lines were kindly donated by Dr Thomas E. Carey, Laboratory of Head and Neck Cancer Biology, University of Michigan. The cell lines were maintained in Dulbecco’s modified Eagle’s medium (DMEM; Invitrogen, Carlsbad. CA, USA) supplemented with 10% fetal bovine serum (FBS; Invitrogen) in a humidified atmosphere of 5% CO_2_ at 37°C.

### Animals

Seven-week-old male athymic nude mice of the KSN strain were purchased from the Shizuoka Laboratory Animal Center (Hamamatsu, Japan) and maintained under specific pathogen-free conditions. Animal experiments were performed with the approval of the Institutional Ethics Committee for Animal Experiments of the Aichi Cancer Center Research Institute.

### Immunohistochemical analysis

One month after the injection of HNSCC cells into nude mice of the KSN strain, the formed subcutaneous tumors were removed and fixed in 10% buffered formalin for 24 h. Formalin-fixed and paraffin-embedded 4-μm sections were used for immunohistochemistry. Following antigen retrieval with a microwave in a citrate buffer (pH 7.4) at 98°C for 10 min and autoclave in 1 mM EDTA (pH 8.0) at 121°C for 5 min, the sections were treated with normal serum to block non-specific reactions and incubated with primary antibodies at optimal dilution at room temperature for 2 h. After washing with phosphate-buffered saline (PBS), the sections were incubated with secondary antibody for 30 min, washed again with PBS, incubated with streptavidin-peroxidase complex (Vectastain ABC kit; Vector Laboratories, Burlingame, CA, USA) for 60 min and counterstained with Mayer’s hematoxylin.

### Growth inhibition assay

The growth inhibitory effect of gemcitabine and/or cetuximab was assessed using Cell Count Reagent SF (Nacalai Tesque, Kyoto, Japan) as a colorimetric indicator for living cells. UM-SCC-6 and UM-SCC-23 cells were seeded in the wells of a 96-well plate and incubated at 37°C under a 5% CO_2_ atmosphere for 24 h prior to the cytotoxicity assay and prior to treatment with increasing doses of gemcitabine and/or cetuximab on day 1. After 3–5 days, 10 μl of Cell Count Reagent SF solution containing WST-8 (5 mM), a colorimetric indicator, was added to each well to evaluate cell viability. The cells were incubated with WST-8 for 3 h. Absorbance in each well was measured at 450 nm using a microplate reader. The cell viability was evaluated using the equation: $({\text{Abs}}_{\text{samples to be tested}}-{\text{Abs}}_{\text{blank}})/({\text{Abs}}_{\text{control}}-{\text{Abs}}_{\text{blank}})\times 100$, where Abs denotes absorbance in each well at 450 nm.

### Cell cycle analysis by flow cytometry

To determine the percentage of cells in various phases of the cell cycle, exponentially proliferating cells were treated with gemcitabine and/or cetuximab. Treated cells and untreated controls were then analyzed for nuclear DNA after propidium iodide staining using CycleTEST™ Plus kit (Becton Dickinson, San Jose, CA, USA) according to the manufacturer’s instructions. Flow cytometric analysis was performed with the FACSCalibur flow cytometer (Becton Dickinson).

### Immunoblotting

Cells were maintained in DMEM supplemented with 10% FBS in 60-mm dishes prior to being lysed at 4°C in lysis buffer [10 mM Tris-HCl (pH 7.5), 150 mM NaCl, NP-40, 1 mM EDTA and Complete Protease Inhibitor Cocktail]. The protein concentration was determined by the Lowry assay (DC Protein Assay; Bio-Rad, Hercules, CA, USA). Protein was resolved by SDS-PAGE and analyzed by western blot analysis using polyvinylidene difluoride membranes according to the manufacturer’s instructions. The membranes were blocked with 5% skimmed milk in Tris-buffered saline (TBS) plus 0.1% Tween-20. The membranes were probed with antibody at 1:1000 dilution in TBS plus 0.1% Tween-20. Equal loading of samples was confirmed by probing the membranes with β-actin antibody (Sigma-Aldrich, St. Louis, MO, USA). The antibodies used were: mouse monoclonal antibody to total EGFR (BD Biosciences Pharmingen/Transduction Laboratories, Franklin Lakes, NJ, USA) and rabbit polyclonal antibodies to total Akt and phospho-Akt (Ser473) (Cell Signaling Technology, Beverly, MA, USA).

### Apoptosis assay

Apoptosis was quantified by measuring caspase 3/7 activity using the Apo-ONE Homogenous Caspase-3/7 assay kit (Promega, Madison, WI, USA). Cells were harvested with trypsin/EDTA and plated at a density of 1×10^4^ cells/96-well plastic plate in DMEM supplemented with 10% FBS on day 0. This was followed by treatment with gemcitabine and/or cetuximab on day 1 in the presence of 10% FBS for 24 h. An aliquot of caspase 3/7 reagent was then added to each well while the plate was agitated for 1 h at room temperature with light protection and subsequently measured for fluorescence intensity by a fluorescent plate reader (Wallac Oy, Turku, Finland).

## Results

### Expression of EGFR in HNSCC cell lines

**Figure 1:**
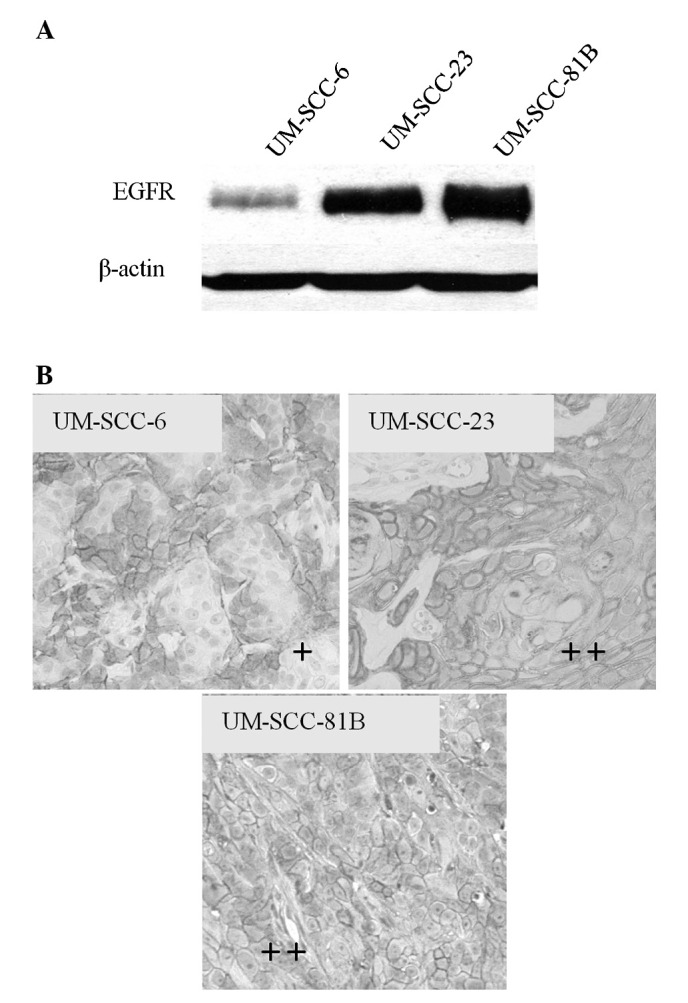
Expression of epidermal growth factor receptor (EGFR) in head and neck squamous cell carcinoma cell lines. Expression of EGFR protein in UM-SCC-6, UM-SCC-23 and UM-SCC-81B cells was assessed by (A) western blot analysis and (B) immunohistochemistry. Actin was used as an internal control.

We investigated two HNSCC cell lines for EGFR protein expression using western blot analysis. The UM-SCC-6 cells exhibited moderate levels and the UM-SCC-23 cells high levels of EGFR protein expression. In addition, the UM-SCC-81B HNSCC cell line was blotted as a candidate for comparison (Fig. 1A). In immunohistochemical analysis, the HNSCC cell lines exhibited identical expression patterns as in western blot analysis (Fig. 1B).

### Growth inhibition effect of cetuximab or gemcitabine

**Figure 2:**
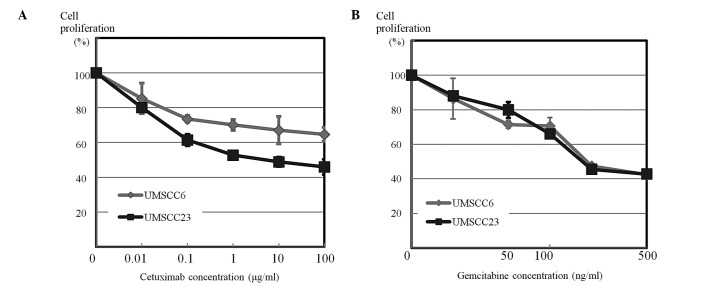
Growth inhibitory effect of cetuximab and gemcitabine on UM-SCC-6 and UM-SCC-23 cell lines. The two cell lines were treated with increasing concentrations of (A) cetuximab (0.01–100 μg/ml) and (B) gemcitabine (20–500 ng/ml). Growth inhibition analysis was performed by the MTT assay. Each data point is the mean of three independent experiments. The vertical bars show standard deviation.

The respective sensitivity of UM-SCC-6 and UM-SCC-23 cells to cetuximab and gemcitabine was determined by the MTT assay and cytotoxicity was found to be concentration-dependent (Fig. 2).

### Growth inhibitory effects of gemcitabine and cetuximab combination treatment on HNSCC cell lines

To investigate the respective sensitivity of UM-SCC-6 and UM-SCC-23 cells to cetuximab and gemcitabine combination treatment *in vitro*, HNSCC cells were treated for 2 h with increasing concentrations (50–500 ng/ml) of gemcitabine and/or for 72 h with a low or high concentration (10 or 100 μg/ml) of cetuximab.

**Figure 3:**
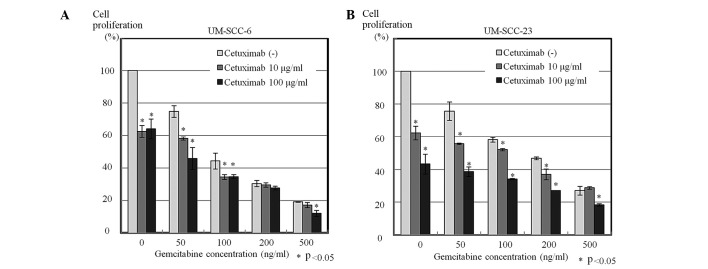
Growth inhibitory effect of gemcitabine and cetuximab combination treatment in head and neck squamous cell carcinoma (HNSCC) cell lines. HNSCC cells were treated with gemcitabine and cetuximab combination and an MTT assay was performed. (A) UM-SCC-6 and (B) UM-SCC-23 cells. Each data point represents the mean of three independent experiments. The vertical bars show standard deviations.

The cell viability in response to gemcitabine alone (50 ng/ml) was found to be 75 and 76% in UM-SCC-6 and UM-SCC-23 cells, respectively. The cell viability in response to gemcitabine (50 ng/ml) and cetuximab (100 μg/ml) was 46 and 39% in UM-SCC-6 and UM-SCC-23 cells, respectively. These results confirmed that gemcitabine and cetuximab in combination exerted an enhanced growth inhibitory effect (Fig. 3).

### Effect of gemcitabine on EGFR and EGFR downstream signaling in HNSCC cell lines

**Figure 4:**
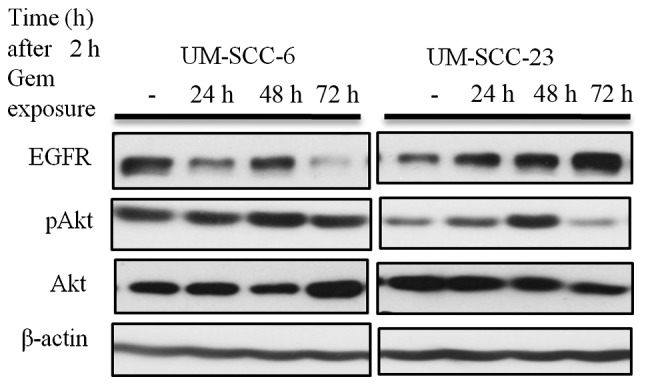
Effect of gemcitabine (Gem) on epidermal growth factor receptor (EGFR) and EGFR downstream signaling in head and neck squamous cell carcinoma cell lines. The effect of gemcitabine on EGFR and EGFR downstream signaling was analyzed by western blot analysis. The cells were exposed to gemcitabine for 2 h after culturing in a normal medium for 72 h. β-actin was used as an internal control.

To elucidate the mechanism of sensitization to gemcitabine following cetuximab pretreatment, the mechanism by which gemcitabine downregulates EGFR or inhibits the EGFR downstream signaling was investigated by western blot analysis. In Fig. 4, EGFR was downregulated 72 h following the exposure of UM-SCC-6 cells to gemcitabine. In addition, EGFR was upregulated time-dependently in UM-SCC-23 cells. Akt was phosphorylated 48 h following gemcitabine exposure but was not inhibited by gemcitabine within 72 h of exposure. Although previous studies have reported that gemcitabine downregulates EGFR (22) or inhibits the EGFR downstream signaling by inhibiting the phosphorylation of Akt (21), no such results were observed in the cell lines examined in the present study.

### Cell cycle distribution of HNSCC cells following gemcitabine and/or cetuximab treatment

**Figure 5:**
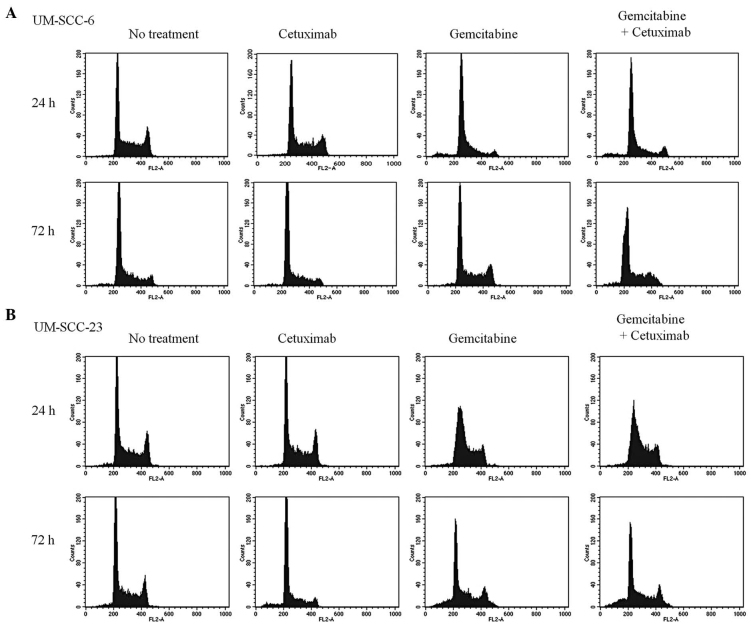
Cell cycle distribution of head and neck squamous cell carcinoma cells following gemcitabine and/or cetuximab treatment. Cell cycle distribution was analyzed by flow cytometry. (A) UM-SCC-6 and (B) UM-SCC-23 cells. Upper panel, 24 h after treatment. Lower panel, 72 h after treatment. From left to right: no treatment, cetuximab 100 μg/ml, gemcitabine 50 ng/ml and gemcitabine 50 ng/ml after cetuximab 100 μg/ml.

To elucidate the mechanism of sensitization to gemcitabine following cetuximab pretreatment, we analyzed the cell cycle distribution following gemcitabine and/or cetuximab treatment. After treatment with gemcitabine alone, the number of cells in the early S-phase was increased at 24 h in the UM-SCC-6 cell line, whereas the same distribution in the control required 72 h. Furthermore, the number of cells in the S phase was significantly increased at 24 h in the UM-SCC-23 cell line and the number of G1 and sub-G1 cells was increased at 72 h. It was hypothesized that the difference in the results between the two cell lines were due to the differences in the mitotic time of each cell type. The cells were accumulated in the S phase following gemcitabine treatment. By contrast, no significant change was observed at 24 h after cetuximab treatment, although the G1 cell population was markedly increased at 72 h. As regards gemcitabine administration after cetuximab, no significant change was observed at 24 h compared to gemcitabine alone; however, after 72 h, the number of sub-G1 phase cells was increased in the two cell lines following combination treatment (Fig. 5).

### Apoptosis was induced by gemcitabine after cetuximab treatment

**Figure 6:**
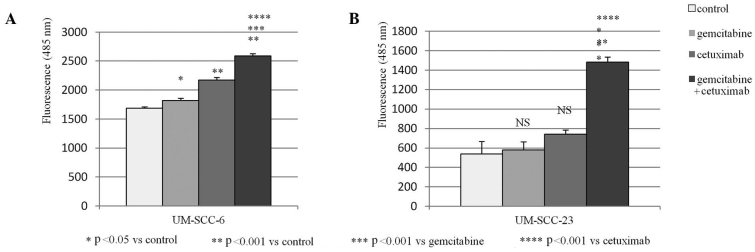
Effects of gemcitabine and/or cetuximab on apoptosis evaluated by measuring caspase 3/7 activity. Head and neck squamous cell carcinoma cells were treated with gemcitabine 50 ng/ml and/or cetuximab 100 μg/ml and evaluated by measuring caspase 3/7 activity.

To analyze the hypothesis that apoptosis is induced to a higher degree by cetuximab compared to gemcitabine treatment, caspase 3/7 activity was measured. In UM-SCC-6 cells, caspase 3/7 activity exhibited a minor increase following single-agent treatment compared to controls, whereas cells treated with gemcitabine after cetuximab exhibited an increase of ~1.5 times compared to controls. Similarly, in UM-SCC-23 cells, no significant difference was observed in controls or cells receiving single-agent treatment. By contrast, the cells treated with gemcitabine after cetuximab exhibited activity ~3 times higher compared to that of the controls (Fig. 6).

## Discussion

The combination therapy of cetuximab with cisplatin, TPF or RT anticancer drugs was previously investigated in HNSCC (7,11–13). However, in cases of residual disease or recurrence after cisplatin, TPF and RT are often selected as the first-line treatment, since the treated cells have likely acquired resistance to cisplatin, 5-FU and DTX. In this study, we investigated the use of cetuximab in combination with other anticancer drugs, excluding cisplatin, 5-FU and DTX.

First, we examined the use of gemcitabine with cetuximab. Gemcitabine is a nucleotide analog classified as an antimetabolite (14), similar to 5-FU, which is one of the most frequently used anticancer drugs for HNSCC. Several studies previously demonstrated that gemcitabine is a treatment choice that is effective against HNSCC (17–19).

In the present study, we confirmed the growth inhibitory effect of gemcitabine against HNSCC cells *in vitro* in a concentration-dependent manner. Similar to gemcitabine, cetuximab also exhibited cytotoxicity against HNSCC cells in a concentration-dependent manner. In addition, gemcitabine and cetuximab combination treatment achieved an enhanced growth inhibitory effect.

It was reported that the combination of gemcitabine with an EGFR-targeting drug was effective against pancreatic carcinoma (20). Moreover, gemcitabine + gefitinib treatment was reported to be effective against HNSCC *in vitro*(21). In the present study, we also confirmed the effectiveness of gemcitabine and cetuximab (but not gefitinib) combination therapy against HNSCC cells. The mechanism of action was found to be the disruption of the cell cycle and/or inhibition of the EGFR downstream signaling. Subsequently, we analyzed the cell cycle distribution by flow cytometry.

It is well-known that gemcitabine exerts its maximum cytotoxic effect on cells in the S phase (15), whereas cetuximab causes cell cycle arrest in the G1 phase (10). Thus, we hypothesized that the greatest synergy would be achieved if gemcitabine was administered prior to cetuximab.

In our series, cells accumulated in the S phase within 24 h following gemcitabine treatment and G1 arrest occurred within 72 h following cetuximab treatment. An increase in the number of sub-G1 phase cells was also observed in the cell lines treated with the two drugs at 72 h. In an apoptosis assay, caspase 3/7 activity was found to be higher with concurrent administration of gemcitabine and cetuximab compared to the administration of either agent alone. These results suggest that the cell cycle disruption was enhanced by the combined cytotoxicity of gemcitabine and cetuximab.

On the basis of this mechanism, we concluded that gemcitabine and cetuximab combination treatment is effective against HNSCC. In a previous study, the downstream signaling of EGFR was inhibited in pancreatic cancer cells by gemcitabine and cetuximab combination treatment (23), which was not observed in the HNSCC cell lines used in our study (Fig. 4). Moreover, repeated gemcitabine treatment was shown to achieve further EGFR degradation in HNSCC cells (22). In our study, the EGFR expression was decreased in UM-SCC-6 but not in UM-SCC-23 cells (Fig. 4), suggesting that this phenomenon may be limited to particular cell lines.

In conclusion, gemcitabine and cetuximab are effective drugs against HNSCC and an enhanced antitumor effect may be expected when using gemcitabine in combination with cetuximab. A recent pilot study that investigated the administration of cetuximab concomitantly with gemcitabine and radiotherapy in advanced squamous cell carcinoma of the upper aerodigestive tract, reported that the toxicity was significant (24). However, the combination of gemcitabine with cetuximab was sufficiently effective in HNSCC *in vitro* and regimens of cetuximab administered concomitantly with gemcitabine and radiotherapy are highly likely to cause side effects. Therefore, further *in vivo* investigation and clinical studies are recommended.
